# Programming of metabolic effects in C57BL/6JxFVB mice by in utero and lactational exposure to perfluorooctanoic acid

**DOI:** 10.1007/s00204-015-1488-7

**Published:** 2015-04-01

**Authors:** J. C. J. van Esterik, L. Bastos Sales, M. E. T. Dollé, H. Håkansson, M. Herlin, J. Legler, L. T. M. van der Ven

**Affiliations:** 1 Center for Health Protection, National Institute for Public Health and the Environment (RIVM), PO Box 1, 3720 BA Bilthoven, The Netherlands; 2 Department of Chemistry and Biology, Institute for Environmental Studies (IVM), VU University, De Boelelaan 1085, 1081 HV Amsterdam, The Netherlands; 3 Institute of Environmental Medicine, Karolinska Institutet, P.O. Box 210, SE-171 77 Stockholm, Sweden

**Keywords:** Endocrine disrupting compounds, Developmental exposure, Metabolic effects, Perfluorooctanoic acid, Programming

## Abstract

Perfluorooctanoic acid (PFOA) is known to cause developmental toxicity and is a suggested endocrine disrupting compound (EDC). Early life exposure to EDCs has been implicated in programming of the developing organism for chronic diseases later in life. Here we study perinatal metabolic programming by PFOA using an experimental design relevant for human exposure. C57BL/6JxFVB hybrid mice were exposed during gestation and lactation via maternal feed to seven low doses of PFOA at and below the NOAEL used for current risk assessment (3–3000 µg/kg body weight/day). After weaning, offspring were followed for 23–25 weeks without further exposure. Offspring showed a dose-dependent decrease in body weight from postnatal day 4 to adulthood. Growth under high fat diet in the last 4–6 weeks of follow-up was increased in male and decreased in female offspring. Both sexes showed increased liver weights, hepatic foci of cellular alterations and nuclear dysmorphology. In females, reductions in perigonadal and perirenal fat pad weights, serum triglycerides and cholesterol were also observed. Endocrine parameters, such as glucose tolerance, serum insulin and leptin, were not affected. In conclusion, our study with perinatal exposure to PFOA in mice produced metabolic effects in adult offspring. This is most likely due to disrupted programming of metabolic homeostasis, but the assayed endpoints did not provide a mechanistic explanation. The BMDL of the programming effects in our study is below the current point of departure used for calculation of the tolerable daily intake.

**Electronic supplementary material:**

The online version of this article (doi:10.1007/s00204-015-1488-7) contains supplementary material, which is available to authorized users.

## Introduction

Perfluorooctanoic acid (PFOA) is a man-made compound that, along with other perfluorinated organic compounds, is able to repel both water and oils. PFOA has many applications, including coatings for fabrics and carpets, food packaging paper products, fire-fighting foams, mining and oil well surfactants and floor polishes (Prevedouros et al. 2006). PFOA is resistant to degradation due to the carbon–fluorine bond, and this stability has led to its widespread use and is also responsible for its persistence in the environment. Although not bioaccumulative (Conder et al. 2008), PFOA has an estimated elimination half-life in humans of around 4 years (Olsen et al. 2009) and has been detected globally in adult human serum samples at 3.6–4.3 ng/mL (Calafat et al. 2007). Similar or lower levels were measured in serum of pregnant mothers at 2.6–4.0 ng/mL (Grandjean et al. 2012) and in cord plasma at 0.3–2.7 ng/mL (de Cock et al. 2014). Dietary exposure, the main exposure route for humans, has been established to be below the tolerable daily intake (TDI) of 1.5 µg/kg bw/d (EFSA 2012). However, uncertainties remain about the developmental effects of PFOA (EFSA 2008; Grandjean and Budtz-Jorgensen 2013).

Fetal and neonatal exposure occurs because PFOA can cross the placenta and is excreted in breast milk (Apelberg et al. 2007a; Henderson and Smith 2007). Animal studies have shown that in utero and lactational exposure to PFOA could lead to developmental effects such as delayed eye opening and a decrease in body weight (Lau et al. 2006), neurotoxicity (Johansson et al. 2008; Mariussen 2012) and mammary gland developmental abnormalities (Macon et al. 2011; White et al. 2011b). PFOA also seems to have the ability to interfere with endocrine systems (Jensen and Leffers 2008; White et al. 2011a). PFOA is suggested to induce estrogen receptor transactivity and antagonize androgen receptor activity in in vitro studies, although this depends on experimental conditions (Kjeldsen and Bonefeld-Jorgensen 2013). Furthermore, PFOA is particularly known to affect metabolism through activation of peroxisome proliferator-activated receptors (PPARs; Abbott et al. 2012).

Early life exposure to endocrine disrupting compounds (EDCs) has been suggested to program the developing organism for chronic diseases later in life, such as metabolic disorders (Baillie-Hamilton 2002). Epidemiological studies have found an association between maternal PFOA serum and reduced birth weight (Apelberg et al. 2007b; Fei et al. 2007; Maisonet et al. 2012), a known risk indicator for metabolic diseases later in life (Ravelli et al. 1998), but others found no association (Hamm et al. 2010; Washino et al. 2009). Moreover, a recent systematic review has concluded developmental PFOA exposure results in decreased fetal growth in both human and non-human mammalian species (Lam et al. 2014). Furthermore, an epidemiological study shows associations of maternal PFOA concentration with increased BMI and waist circumference, serum insulin and leptin levels, and lower adiponectin levels, particularly in female offspring at the age of 20 years (Halldorsson et al. 2012). However, a study with a wider range of estimated PFOA exposure early in life did not find an association with the risk for overweight or obesity in adulthood (Barry et al. 2014). In one animal study described by Hines et al. (2009), gestational exposure of mice to relatively low doses of PFOA resulted in an increase in body weight and insulin and leptin levels during midlife in female offspring. Programming by EDCs for chronic diseases later in life can occur through changes in the epigenome, especially the DNA methylation pattern (Barres and Zierath 2011; Ruchat et al. 2013). For example, an epidemiological study shows global hypomethylation in umbilical cord serum associated with PFOA exposure (Guerrero-Preston et al. 2010).

Given the limited and inconsistent existing data on the metabolic programming effects of PFOA, the goal of the present study was to test the hypothesis that early exposure to low doses of PFOA can program mice for the development of metabolic impairment later in life. We aimed to model human exposure conditions closely, through use of a broad exposure window of both gestational and lactational exposure and by mimicking human dietary exposure via maternal feed. We applied a dose–response design, using a control group and a dose range of 3–3000 µg/kg body weight/day (µg/kg bw/d). Finally, after a latency period of 23–25 weeks, the adult metabolic phenotype of the offspring was analyzed. In the final part of the study, offspring were challenged with a high fat diet to test whether their sensitivity to energy-dense feed was affected.

## Methods

### Test chemical and test diets

PFOA was obtained as sodium perfluoro-n-octanoate (PFOA-Na; CAS No. 335-95-5, purity >99 %) and kindly provided by Wellington Laboratories Inc., Ontario, Canada. PFOA-Na was dissolved in acetone and mixed with the diet (NIH-07, Research Diet Services, Wijk bij Duurstede, The Netherlands). Thereafter, acetone was allowed to evaporate, and this master mixture was serially diluted with a factor 3–3.3 by adding NIH-07, and afterward, the diet was pelleted. In this way, seven diet groups and a control NIH-07 (blank acetone was added) were obtained, aiming at concentrations of 0, 0.017, 0.056, 0.17, 0.56, 1.7, 5.6 and 17 mg/kg PFOA in feed, which corresponded to 0, 3, 10, 30, 100, 300, 1000 and 3000 µg/kg bw based on an average food consumption of 4.5 g per mouse (average body weight of 25 g) per day. PFOA concentrations in test diets were confirmed by isotope dilution liquid chromatography mass spectrometry after extraction with methanol (8402, JT Baker, Deventer the Netherlands) and a cleanup with carbon solid phase extraction (ENVI Carb, 57210-U, Supelco, Zwijndrecht, the Netherlands). Actual PFOA concentrations in the food were 15–30 % lower than the nominal values. The non-purified soy-based NIH-07 was chosen because it was originally designed to optimize gestation, lactation and growth of rodents, and avoiding natural phytoestrogens in the diet may have confounding effects (Ruhlen et al. 2008). Estrogenic and anti-estrogenic activity for this diet was previously analyzed to be low and absent, respectively (van Esterik et al. 2014).

A high fat diet (D12451, Research Diet Services, Wijk bij Duurstede, The Netherlands), containing 45 kcal % fat (lard) compared with 15 kcal % fat in the NIH-07 diet, was given to all F1 mice during the final weeks of the study (21–28 weeks of age).

### Experimental conditions

Nulliparous female C57BL/6J mice (Charles River, Sulzfeld, Germany) were mated with male FVB mice (GPL, Bilthoven, The Netherlands) to produce hybrid offspring, for which comprehensive background information of phenotype and development is available (Dollé et al. 2011). Mice were maintained under specific pathogen-free conditions with a target ambient temperature of 21 °C, humidity of 60 % and with a 12/12 h light/dark cycle. F0 males were single-housed in standard Macrolon type II cages and were fed standard laboratory chow (CRM, Tecnilab-BMI, Someren, The Netherlands). To avoid interference from environmental bisphenol A (van Esterik et al. 2014), F0 females and their pups during lactation were housed in polysulfone cages (Tecnilab-BMI, Someren, The Netherlands), and drinking water was supplied in glass bottles with rubber stoppers. Cages had spruce/fir wood bedding (Lignocel S 8-15; Tecnilab-BMI, Someren, The Netherlands) and aspen wood shavings (Lignocel 9 S) for cage enrichment. Both feed and water were supplied ad libitum.

After an acclimatization period of 1 week, female F0 mice were fed experimental diets explained above starting 2 weeks before mating and continued during mating (1 week), gestation (3 weeks) and lactation (3 weeks). Each dose group had six F0 females, which were mated three by three with two F0 males for each dose group. We previously observed that 6–9 is a range where litter size does not confound postnatal growth, and therefore, two outlying litters of 5 and 3 pups (dose groups 3 and 3000, respectively) and three litters >9 (dose groups 0, 3, 100) were discarded (van Esterik et al. 2014). For every dose group, on average 9 mice per sex (range 6–10) were included for follow-up through juvenile and adult stages, selecting pups proportionally from available litters. F1 males were single-housed, and F1 females were housed in pairs from the same litter in standard Macrolon type I cages. Body weight was measured at postnatal day (PND) 4, 7, 14 and 21 and weekly from 5 weeks of age continuing until the end of the study. Food consumption could not be recorded reliably due to high spillage.

At the age of 26 weeks (males) and 28 weeks (females), animals were fasted for 18 h to induce a general basic metabolic state and glucose was measured in tail vein blood using the FreeStyle Lite meter and test strips (Abbott, Hoofddorp, The Netherlands). Subsequently, for terminal necropsy, mice were anesthetized by combined treatment with ketamine and xylazine and killed by bleeding. To maximize the yield, blood was extracted from the orbital vasculature. Blood was then treated with Pefabloc SC (PSC) and PSC-Protector solution (Roche, Mannheim, Germany) to neutralize serine proteases. Blood was allowed to clot, centrifuged, and serum samples were stored at −80 °C until further analysis. During necropsy, body length (nose–tail base) was measured and a selection of organs was weighed, including adrenal glands, brain, liver, femur, quadriceps femoris muscle, pancreas, interscapular fat, perigonadal fat and perirenal fat.

This study was approved by the Animal Experimentation Ethical Committee of our institute under permit number 201000078 and carried out in accordance with prevailing legislation.

### In vivo experiments

At 19 weeks of age, a glucose tolerance test (GTT) with fasting for 18 h before the start of the experiment was performed in control and middle dose (300 µg/kg bw/day) males and females as described in van Esterik et al. (2014). One week later, at 20 weeks of age, an insulin tolerance test (ITT) was performed in the same animals as used for the GTT. Mice were not fasted to avoid low glucose baseline levels which, after an insulin injection, could lead to hypoglycemia. At the start of the experiment, a baseline blood sample was taken (0 min). Subsequently, human insulin (Sigma, Zwijndrecht, The Netherlands) was injected intraperitoneally at a concentration of 0.6 IU/kg bw, and glucose was measured in tail vein blood after 15, 30, 45 and 60 min using the FreeStyle Lite meter and test strips (Abbott, Hoofddorp, The Netherlands). The experiment was performed over two morning sessions, with 1.75 h between the first and last tested animal in each session, and animals were treated in a random order. At the age of 22–25 weeks, control and 300 µg/kg bw/d animals were used for a spontaneous locomotor activity test. After an acclimatization period of minimal 6 h, activity of the mice was continuously registered on four parallel platforms for 36 h (males) or 60 h (females), starting at the beginning of the dark phase (6.30 PM) of the first day. Further details are described in van Esterik et al. (2014).

### Ex vivo experiments

For bone analysis, right tibias were cleaned from soft tissue and stored in Ringer solution at −20 °C until analysis. The length was measured using an electronic sliding caliper to the nearest 0.01 mm (IP65, Sylvac SA, Crissier, Switzerland). The tibias were scanned using a peripheral quantitative computed tomography (pQCT) system (Stratec XCT Research SA+) with software version 5.50 (Norland Stratec Medizintechnik, GmbH, Birkenfeld, Germany). The scans of metaphysis and diaphysis were performed at sites distanced 10 and 45 %, respectively, of the length from the growth plate. The thresholds for defining trabecular bone were 280 and 400 mg/cm^3^, while cortical bone was defined above a threshold of 710 mg/cm^3^.

For histopathology, dissected organs were partly or entirely fixed in 4 % formalin for 24 h (except femur), subsequently placed in 70 % alcohol and routinely embedded in paraffin, sectioned and stained with hematoxylin and eosin. Histopathological analysis of the liver comprised an arbitrary semiquantitative scoring of selected marks (eosinophilic alteration, karyomegaly, fatty change). After routine histopathological reading of the sections, adipocyte size and proxy for adipocyte number in perirenal white adipose tissue (WAT) were measured as described in van Esterik et al. (2014). Lipid accumulation in brown adipose tissue (BAT) adipocytes was scored semiquantitatively in the interscapular fat depot. Gene expression of *ucp1*, a marker of energy expenditure through thermogenesis which contributes to regulation of body weight (Kozak et al. 2010), was measured in BAT by qPCR for control and two dose groups (300 and 3000 µg/kg bw/d). *Cidea* is a marker of BAT adipocytes (Zhou et al. 2003) and was used as a normalizer for the contents of BAT adipocytes in the tissue extracts. Relative quantification was performed by the comparative C_T_ method (Schmittgen and Livak 2008). Finally, serum lipids and endocrine parameters were analyzed. Milliplex kits (Millipore Corporation, Billerica, MA) were used according to the manufacturer’s protocol to measure serum adiponectin, ghrelin, glucagon, insulin and leptin. Further technical details for *ucp1* expression and serum lipids are described in van Esterik et al. (2014).

### Statistical analyses

Data were analyzed for statistically significant dose–responses using the benchmark dose (BMD) approach (Slob 2002) with the PROAST software versions 38.0 and 38.1 (www.rivm.nl/proast). In this approach, optimal models from the exponential and Hill families are fitted to data covering the entire study population, and a BMD with its 5 % lower and upper bounds of the 90 % confidence interval (BMDL, BMDU) is derived from the fitted models at a predefined benchmark response (critical effect size, CES). By default, the CES used in this study was 5 % for continuous data, as proposed by the European Food Safety Authority (EFSA 2009). The goodness of fit was determined by the log likelihood of each model within a family of models. The optimal model selected for each family was the model with the lowest number of parameters, which gave the best significant fit. By using the bootstrap method to calculate the 90 % confidence interval surrounding the BMD, individual animals from the same litter were clustered to account for litter effects. In the evaluation of results, data which did not produce a statistically significant dose–response with both exponential and Hill models were not deemed sufficiently informative for robust conclusions. Furthermore, data that produced dose–responses with a wide confidence interval (BMDU/BMDL ratio >100) were not considered suitable to derive a valid benchmark dose.

Growth was calculated by dividing body weight of subsequent weeks by body weight at week 5, i.e., at the start of the F1 follow-up. Weekly maximum effect sizes of growth were derived from the c-parameter of growth dose–response functions, and when no c was available, calculated as a difference between top dose and control (background) values.

Some measurements included only control and one or two dose groups and could therefore not be analyzed as dose–responses. GTT was therefore evaluated by repeated-measures or nested (to account for litter covariance) two-way ANOVAs (Graphpad Prism 5.0, R) to detect differences at the different time points and between the areas under the curve (AUC). Activity measurement, *ucp1* expression, WAT adipocyte size and the proxy for cell number were also tested with a nested ANOVA (R). Differences in distribution of BAT histopathology scores between experimental groups were tested for statistical significance in a two-tailed Fisher’s exact test.

## Results

### General toxicity and reproduction parameters

**Fig. 1:**
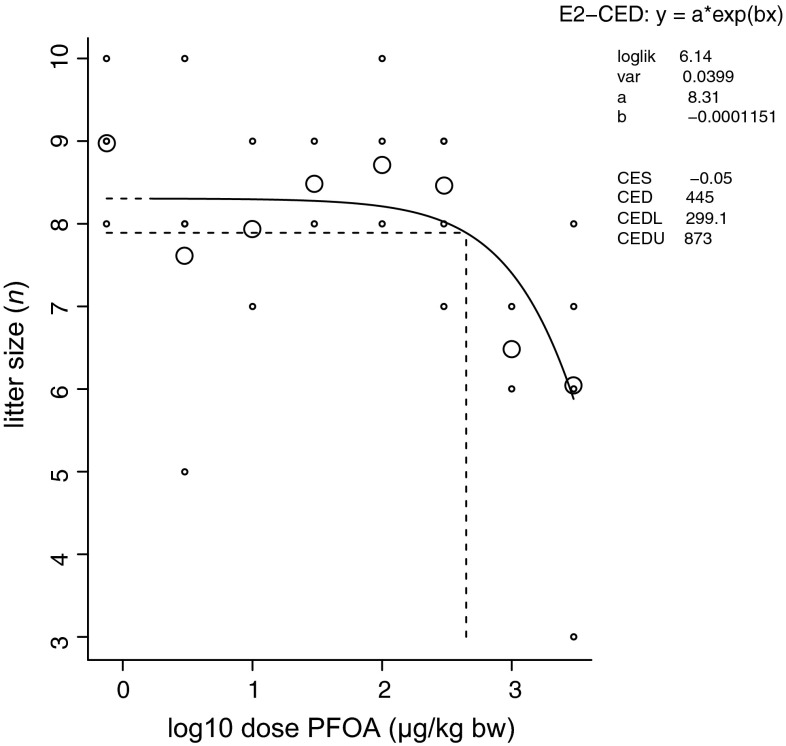
Litter size after perinatal PFOA exposure. F0 C57BL/6J mice were exposed via the diet 2 weeks before mating until postnatal day 21 to PFOA doses of 0–3000 µg/kg body weight/day. Explanation of the dose–response graph is in Fig. 2 legend

In dams, dietary exposure to 3–3000 µg/kg bw/day PFOA had no effect on measures of general toxicity, including survival, body weight and weight gain during gestation (data not shown). The lack of an effect on body weight or body weight gain also supports that PFOA did not adversely affect the palatability of the feed. Average mating success rate was 73 %, yielding 32 litters with an average litter size of 7.9 (range 3–10; Fig. 1). The overall F/M sex ratio in the F1 generation was 0.9, and the overall survival rate was 98 %. No effects of PFOA exposure were found on sex ratio or survival of the F1 generation (data not shown). Maternal predation of pups was observed in three litters, however, without relation to PFOA exposure, and in general, parental behavior was normal. PFOA appeared to have a reprotoxic effect on litter size, with dose groups 1000 and 3000 µg/kg bw being above the BMD of 445 µg/kg bw/d (Fig. 1). For this reason, analyses testing only control and single dose groups were performed at the non-toxic dose of 300 µg/kg bw/d (incidentally also 3000 µg/kg bw/d).

### Body weights

**Fig. 2:**
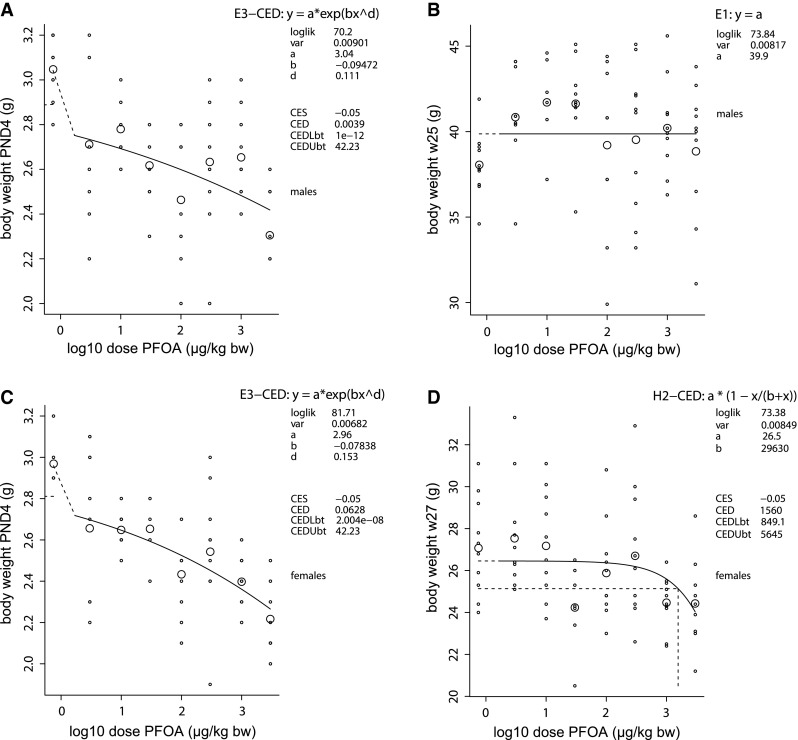
Dose–responses of body weight at early age and in adulthood after perinatal PFOA exposure. C57BL/6JxFVB hybrid mice were perinatally exposed via maternal diet to 0–3000 µg/kg body weight/day PFOA during gestation and lactation. Body weight at early age, postnatal day 4, in **a** males and **c** females. Body weight in adulthood, in **b** males (week 25) and in **d** females (week 27). The function of the curves is shown in the top line in the upper right corner of each graph, followed by parameters of significance and shape of the curve (loglik, var). CES, critical effect size. CED, critical effect dose; CEDLbt, CEDUbt, the lower and upper bound of the (two-sided) 90 % confidence interval for the CED, calculated by the bootstrap method (in the text denoted as BMD, BMDL and BMDU, respectively). *Small symbols* individuals, *large symbols* geometric mean (per dose). The analysis was done with PROAST versions 38.0-38.1

**Fig. 3:**
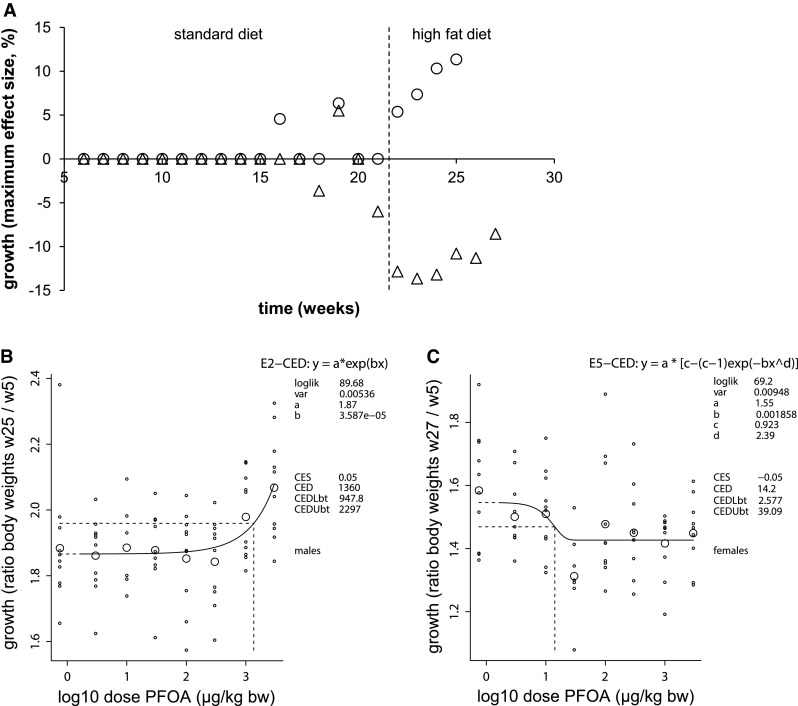
Growth throughout study and representative dose–responses after perinatal PFOA exposure. C57BL/6JxFVB hybrid mice were perinatally exposed via maternal diet to 3000 µg/kg body weight/day (µg/kg bw) PFOA during gestation and lactation. **a** Growth throughout the study, indicated by maximum effect sizes, in males (*circles*) and in females (*triangles*). The *line* represents the change to a high fat diet at week 21. Representative dose–responses for growth in **b** males and **c** females at the end of the study. Growth is calculated by dividing body weight of every week with body weight at the start of the F1 period (week 5). For every week, a growth dose–response was produced and maximum effect size calculated. Explanation of the dose–response graph is in Fig. 2 legend

Males showed a dose-dependent decrease in body weight at PND4 (Fig. 2a), indicating that dosing was received properly despite the lack of measurement of internal dose. This effect persisted during lactation and during the post-exposure period, with a standard diet, until adulthood at 21 weeks of age (Table 1). After the start of the high fat diet regime at week 21, this dose-dependent decrease in body weight was also present in weeks 22 and 23 (data not shown). Under this high fat diet regime (weeks 21–25), however, a PFOA dose-dependent increase in growth occurred (Fig. 3a, b) and concomitantly the dose-dependent decrease in body weight was no longer present in the final 2 weeks of the study (Fig. 2b).

**Table 1:** Summary of dose–response results in offspring after perinatal exposure to PFOA

	Males					Females				
	Dose–response	BMDL (µg/kg bw/d)	BMDU (µg/kg bw/d)	Max effect size (%)	Relative to bw^a^	Dose–response	BMDL (µg/kg bw/d)	BMDU (µg/kg bw/d)	Max effect size (%)	Relative to bw^a^
Body weight										
Week 21	↓	1024	3457	−9.1		↓	1239	3595	−8.0	
Week 25 (m), 27 (f)	–					↓	849	5645	−9.1	
Body size										
Body length	–				–	–				↑
Femur length	–				–	↓	2423	6999	−4.2	↑
Tibia length	↓	ni^b^	ni	−1.6	–	↓	ni	ni	−5.0^c^	↑
Growth										
Week 25/5 (m), 27/5 (f)	↑	948	2383	11		↓	ni	ni	−8.6	
Organ weights										
Adrenal glands	–				–	–				↑
Brain	–				–	–				↑
Femur	–				–	↓	989	5060	−8.6	↑
Liver	↑	ni	ni	16	↑	–				↑
Quadriceps femoris muscle	–				–	↓	432	1356	−19	–
Pancreas	–				–	–				↑
Fat pad weights										
Interscapular	–				–	–				–
Perigonadal	–				–	↓	ni	ni	−56	↓
Perirenal	–				–	↓	65	362	−57	↓
Tibia composition/function^d^										
Cortical density	↓	2635	27,520	−3.4		↓	2866	6949	−3.7	
Ability to resist torsion	–					↓	917	10,316	−8.3	
Bending strength	–					↓	888	3371	−9.9	
Trabecular area	–					↓	914	3037	−10	
Serum lipids, glucose										
Cholesterol	–					↓	402	1284	−20	
Free fatty acids	–					–				
High-density lipoproteins	–					–				
Triglycerides	–					↓	6.2	623	−27	
Glucose	–					–				
Serum hormones										
Adiponectin	–					–				
Ghrelin	–					–				
Glucagon	–					–				
Insulin	–					–				
Leptin	–					–				

↑, ↓—significant increase, decrease dose–responses, or absence of effect. Single sign represents exponential (E) and Hill (H) modeling outcomes. A BMDL (lowest 5 % lower confidence bound of the BMD at a critical effect size of 5 %) and BMDU are only given in case of a small confidence interval (BMDU/BMDL ratios <100); data with a wider confidence interval are not considered informative (ni). A maximum effect size is derived from the c-parameter if present in the selected dose–response models, otherwise calculated as a difference between top dose and control (*background*) values, and the reported value is an average of E and H maximum effect sizes. Organ and fat pad weights were also analyzed relative to body weight to detect interdependency of PFOA and body weight

bw, body weight; m/f, males/females; µg/kg bw/d, µg PFOA/kg body weight/day

^a^All relative to body weight dose–responses for males and females had a BMDU/BMDL ratio >100, and thus, BMDL and BMDU are not informative, except for female liver (BMDL-BMDU: 1130–4452 µg/kg bw/d), pancreas (708–2533 µg/kg bw/d) and perirenal fat (82–435 µg/kg bw/d)

^b^BMDU/BMDL ratio <100, however, BMDL and BMDU are not considered informative since BMDL (6124 µg/kg bw/d) is higher than top dose (3000 µg/kg bw/d)

^c^Dose–response and maximum effect size for tibia length are solely based on exponential modeling, since Hill modeling gave an error

^d^Other parameters for tibia composition/function that did not show a dose–response in either of the sexes were total density, total area, cortical area, cortical thickness, periosteal circumference, endosteal circumference and trabecular density

Similar to males, female body weight was dose-dependently decreased from PND4 (Fig. 2c) until adulthood (week 21, standard diet). This effect continued under a high fat diet until the end of the study, week 27 (Fig. 2d), with a BMDL of 849 µg/kg bw/d (Table 1). Females showed a negative dose–response for growth that started at week 21 under standard diet and persisted under a high fat diet during the final weeks of the study (Fig. 3a, c).

### Organ and fat pad metrics

An overview of dose–responses for organ and fat pad metrics is given in Table 1. In males, none of the organ and fat pad weights showed a dose–response, except for absolute and relative (to body weight) liver weights, which both showed a dose-dependent increase and tibia length, which showed a dose-dependent decrease. Female organs were not affected by PFOA, except for femur and tibia length, femur weight and weight of the quadriceps femoris muscle, which all showed a dose-dependent decrease (Table 1). However, relative to body weight, dose–responses for femur and tibia length and femur weight were positive, and for weight of the quadriceps femoris muscle, dose–responses were absent. Moreover, positive dose–responses of relative organ weights that did not show an effect on absolute organ weights, particularly brain and liver, were observed. Absolute and relative weights of perigonadal and perirenal fat pads showed negative dose–responses (Supplemental Fig. 1), with a BMDL of 65 µg/kg bw/d for perirenal fat pad (Table 1).

In both males and females, the observed negative dose–response for cortical density was weak, indicated by high BMDLs. In addition, in females, the negative dose–responses for the ability to resist torsion, bending strength and trabecular area had BMDLs corresponding to other parameters (e.g., femur weight) and maximum effect sizes of around 10 %. However, for all three parameters, the top dose group showed a small variation compared with the seven other groups, which could be largely responsible for the observed dose–responses.

### Histopathology

**Fig. 4:**
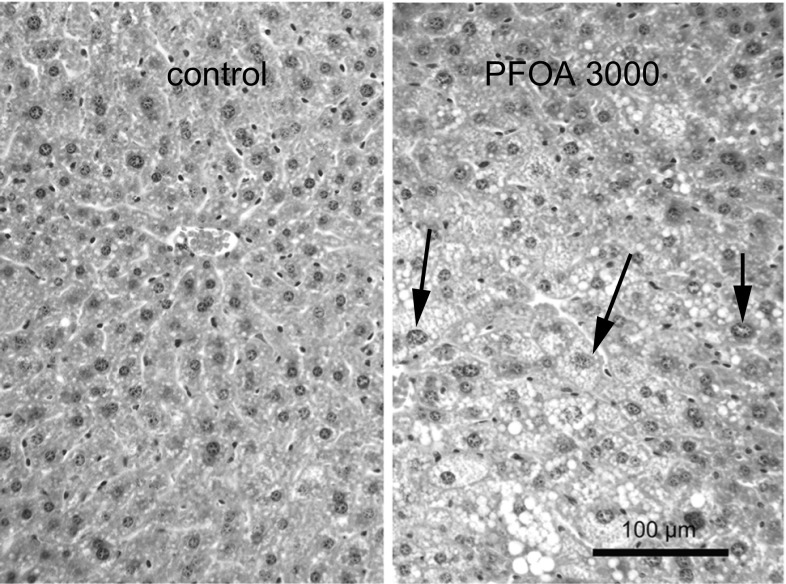
Microphotographs of liver effects in males at 26 weeks after perinatal exposure to PFOA 3000 µg/kg body weight/day (*right*) as compared to control (*left*). C57BL/6JxFVB hybrid mice were perinatally exposed via maternal diet to 3000 µg/kg body weight/day PFOA during gestation and lactation. The figure illustrates lipid accumulation in hepatocytes (microvesicular steatosis, showing as white vesicles) and large nuclei (karyomegaly, *arrows*)

Histopathological analysis of the liver revealed foci of cellular alterations, mostly of an eosinophilic appearance, which were nearly significantly (*p* = 0.07) more frequently observed in the PFOA-exposed males (300 and 3000 µg/kg combined), compared with the control males (Table 2). In females, controls already showed frequent occurrence of such foci and the distribution of grades did not statistically differ between control and PFOA-exposed females. A second observation was nuclear dysmorphology, e.g., the presence of hepatocellular nuclei of varying sizes (anisokaryosis) or of notably large nuclei (karyomegaly), which was a notable mark and nearly statistically significant (both sexes *p* = 0.06) in the PFOA-exposed males and females (Table 2; Fig. 4). An additional observation was PFOA exposure-associated fatty change of hepatocytes, mainly of the microvesicular type (Fig. 4), however, without statistical significant difference of distribution.

**Table 2:** Histopathology grades of liver

	Eosinophilic alteration^a^		Nuclear dysmorphology^b^		Fatty change	
	Grade 0	Grades 1, 2	Grade 0	Grades 1, 2	Grade 0	Grades 1, 2, 3, 4
Males						
Control	10	0	10	0	10	0
PFOA	14	6	13	7	16	4

	Eosinophilic alteration		Nuclear dysmorphology^b^		Fatty change	
	Grades 0, 1	Grades 2, 3	Grade 0	Grades 1, 2	Grade 0	Grades 1, 2, 3
Females						
Control	7	3	9	0	10	0
PFOA	11	8	12	7	17	2

Grades were defined through a first blinded screening of sections and represent a range for focus of eosinophilic alteration (left) from no (grade 0), moderate (grade 1), to strong (grade 3); a range of nuclear dysmorphology, e.g., anisokaryosis/karyomegaly (middle) from no (grade 0), moderate (grade 1), to strong (grade 2) and a range of fatty change (right) from no (grade 0), moderate (grade 2), to strong (grade 4). In this distribution table, numbers are counts of perinatally control and PFOA-exposed (300 and 3000 µg PFOA/kg body weight/day) individual males (upper) and females (bottom) with a given grade. Results for both PFOA dose groups were similar, and data have been combined for statistical power

^a^The distribution in the PFOA-exposed males versus control males is nearly statistically significant (*p* = 0.07) in a two-tailed Fisher’s exact test

^b^The distribution in the PFOA-exposed animals versus control animals in both males and females is nearly statistically significant (for both *p* = 0.06) in a two-tailed Fisher’s exact test

**Fig. 5:**
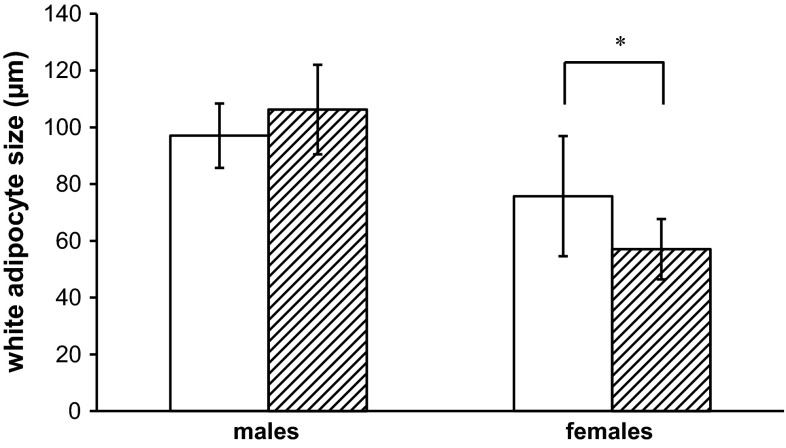
White adipocyte size (measured in perirenal fat pad) after perinatal PFOA exposure in males and females. C57BL/6JxFVB hybrid mice were perinatally exposed via maternal diet to PFOA during gestation and lactation. Data reflect control animals (*open bars*) and two PFOA dose groups, 300 and 3000 µg PFOA/kg body weight/day (*striped bars*), which showed similar results and were combined for nested ANOVA analysis. *The difference in white adipocyte size between PFOA-exposed females and control females is statistically significant (*p* < 0.05) in a nested ANOVA analysis

Histopathology of brown adipose tissue (interscapular fat pad) showed significant lipid accumulation in the PFOA-exposed males compared with control males (Table 3). For females, a trend for lipid depletion in PFOA-exposed females was observed (Table 3), but the distribution between PFOA-exposed females and control females did not statistically differ (*p* = 0.097). However, in white adipose tissue, both adipocyte cell size (measured in perirenal fat pad; Fig. 5) and cell number (data not shown) showed no differences between PFOA-exposed males and control males, while for females, cell size was significantly decreased in PFOA-exposed animals compared with controls and cell number did not differ (data not shown).

**Table 3:** Histopathology grades of brown adipose tissue

	Males^a^		Females^b^	
	Grades 1, 2, 3	Grades 4, 5, 6	Grades 1, 2	Grade 3
Control	7	3	4	6
PFOA	4	13	14	4

Grades were defined through a first blinded screening of sections and represent a range of lipid accumulation from no (grade 1), moderate (grade 3), to strong (grade 6) lipid accumulation. In this distribution table, numbers are counts of perinatally control and PFOA-exposed (300 and 3000 µg PFOA/kg body weight/day) individuals with a given grade. Results for both PFOA dose groups were similar, and data have been combined for statistical power

^a^The distribution in the PFOA-exposed males versus control males is statistically significant (*p* < 0.05) in a two-tailed Fisher’s exact test

^b^For females, the distribution between controls and PFOA-exposed females did not statistically differ but shows a trend (*p* = 0.097)

### Metabolic phenotype

To explain the change in body and organ weights, other metabolic and energy balance-related parameters were analyzed in one (300 µg/kg) or two (300 and 3000 µg/kg) dose groups and control animals, including glucose homeostasis [GTT, ITT, basal glucose (measured in all dose groups, Table 1)] and energy expenditure (physical activity, *ucp1* expression in brown adipose tissue for temperature regulation). In both males and females, PFOA did not show an effect on any of these parameters (data not shown).

To further explore the metabolic phenotype, both lipid (cholesterol, free fatty acids, high-density lipoproteins and triglycerides) and endocrine parameters (leptin, ghrelin, adiponectin, insulin, and glucagon) were measured in serum. In males, none of these serum parameters were affected. However, in females, both cholesterol and triglycerides showed a dose-dependent decrease (Supplemental Fig. 2) with a BMDL of 402 and 6.2 µg/kg bw/d, respectively (Table 1).

## Discussion

We studied whether early exposure to low doses of PFOA could program C57BL/6JxFVB hybrid mice for metabolic impairment later in life. Offspring showed a dose-dependent decrease in body weight, which already existed early in life (PND4) and was maintained in adulthood. Furthermore, growth under standard diet in both sexes was not affected. Therefore, this can be understood as a direct developmental effect at the highest doses. In contrast, other developmental programming studies summarized in Table 4 show a body weight decrease in male and/or female pups, occurring during lactation but without follow-up or persistence into adulthood. Also, incidental body weight increases were observed in later life, although under different experimental conditions (Hines et al. 2009; Wolf et al. 2007; details Table 4). In our study, high fat diet countered the PFOA dose-related reduction in body weight in males, leading to a positive dose–response for growth during the high fat diet regime, and hence, no observed effect in body weight for males during the final 2 weeks of the study. The persistent decrease in body weight of our females until adulthood was worsened by a dose-dependent decreases in growth starting at the last week under standard diet and continuing under high fat diet.

**Table 4:** Effects of early life exposure to PFOA in mice studies

Strain	Dose (µg/kg bw/d)	Diet	Exposure route	Exposure window	Endpoint bw (age)		Time of effect^a^	Other relevant effects of PFOA	References
					M	F			
CD-1	1000; 3000; 5000; 10,000; 20,000; 40,000	LabDiet 5001; PMI Nutrition international LLC	Oral gavage	GD1-17	↓ PND1-25; – 6.5w-60w	↓ PND1-25; – 6.5w-60w	T	M: earlier preputial separation	Lau et al. (2006)
CD-1	10; 100; 300; 1000; 3000; 5000	LabDiet 5001; PMI Nutrition international LLC	Oral gavage	GD1-17	nd	↓ PND1,21 (high doses); ↑ 20-40w (low doses)	D T	21–33w: ↑ insulin; ↑ leptin (low doses); 18 m, weights abs/rel: ↓ abdominal white fat (high doses); ↑ interscapular fat (high doses); – liver	Hines et al. (2009)
CD-1	5000	LabDiet 5001; PMI Nutrition international LLC	Oral gavage	GD1-17; GD8-17; GD12-17	↓ PND1-20	↓ PND1-20	P^c^	altered mammary gland development in dams + female pups	White et al. (2007)
CD-1	3000; 5000	LabDiet 5001; PMI Nutrition international LLC	Oral gavage	GD1-17	nd	↓ PND21-85	T	weaning age: abnormal (delay) mammary gland development; ↑ rel liver weight	White et al. (2009)
CD-1	1000; 5000; 0/1000 + 5 ng/mL via drinking water	LabDiet 5001; PMI Nutrition international LLC	Oral gavage	GD1-17; GD1-end of study (5 ng/mL groups)	nd	↓ PND42 (F1); ↑ PND14-22 (F2)	T	delay in mammary gland development and/or lactational differentiation (P0, F1, F2)	White et al. (2011b)
CD-1	3; 5	LabDiet 5001; PMI Nutrition international LLC	Oral gavage	GD1-17^b^	↓ PND1-22; ↑ PND85 - 35w (dose 3000)	↓ PND1-22	T; D (dose 3000)	M + F, PND22: ↑ abs/rel liver weight	Wolf et al. (2007)
NMRI	580-8,700	standardized pellets (llLactamin, Sweden)	Oral gavage	PND10	– PND10 + 28	nd	–	8/16w: neurotoxicity	Johansson et al. (2008)
Balb/c and C57BL/6	1000-5000-10,000	8640 Harlan Teklad 22/5 Rodent Diet	Oral gavage	PND21-49 (5d/w)	nd	↓ PND45-46	E	PND49: delayed vaginal opening; ↑ abs/rel liver weight; inhibition mammary gland/uterine development	Yang et al. (2009)
C57BL/6	5000	8640 Harlan Teklad 22/5 Rodent Diet	Oral gavage	PND21-49 (5d/w)	nd	nd	–	↑ serum progesterone; ↑ mammary gland response to estrogen and/or progesterone	Zhao et al. (2010)
Balb/c; C57BL/6	2500 (Balb); 7500 (BL6)	8640 Harlan Teklad 22/5 Rodent Diet	Oral gavage	PND21-49 (5d/w)	nd	↓ PND42-49 (BL6)	E	delayed/absent vaginal opening; lack of estrous cycling; ↓ovarian steroid hormonal synthetic enzyme levels	Zhao et al. (2012)
C57BL/6/Bkl	300	not specified	Diet	GD1-PND0	nd	nd	–	M + F: changes in exploratory behavior; M: ↑ activity	Onishchenko et al. (2011)
CD-1	300-1000-3000	not specified	Oral gavage	GD1-17	– PND7-85	– PND7-85	–	M + F: delayed mammary gland development	Macon et al. (2011)
CD-1	10-100-1000	not specified	Oral gavage	GD10-17	– PND1-21	– PND1-21	–	M + F: hepatomegaly; F: mammary gland developmental abnormalities	
CD-1	10-100-1000	NIH-31	Oral gavage	PND18-20	nd	– PND21	–	PND21: ↑ abs/rel uterine weight (lowest dose)	Dixon et al. (2012)
C57BL/6JxFVB	3-10-30-100-300-1000-3000	NIH-07; HFD (D12451) w21-27	Diet	2w pre-mating-PND21	↓ PND4–w23	↓ PND4–w27	P^d^	F: ↓ fat pad weights; ↓ serum cholesterol; ↓ serum triglycerides	This study

abs/rel, absolute/relative; Balb, Balb/c; BL6, C57BL/6; bw, body weight; m, months; w, week(s); GD/PND, gestation/postnatal day; HFD, high fat diet; nd, no data; M/F, male/female; µg/kg bw/d = µg PFOA/kg body weight/day

^a^P, permanent effect on body weight throughout study; D, delayed (late onset) of effect; T, transient effect; E, effect observed during treatment

^b^Several shorter exposure windows within GD1-17 were also tested

^c^No follow-up of animals; similar study of White et al. (2009) with follow-up indicates body weight effect is transient

^d^In males, effect was not observed during final 2 weeks of the study (w24–25), when animals were fed a high fat diet

The reduced femur and tibia length and weights of the femur and quadriceps femoris muscle in females also suggest that body size was affected. The ratios of the femur parameters to body weight showed a positive dose–response, however, indicating that body weight was more severely affected than body size. This is supported by a positive dose–response of relative weights of organs that showed no effect of absolute weights, particularly brain and liver. In contrast, relative weights of the perigonadal and perirenal fat pads maintained a negative dose–response, as did the absolute weights. This indicates that relative weight decrease in these organs is stronger than the decrease in total body weight, and the decrease in fat mass may thus provide a large contribution to the decrease in body weight. A decrease in white fat mass was also observed by Hines et al. (2009) at 1-5 mg PFOA/kg bw/d. This decrease in fat mass could be explained by a decrease in white adipocyte size, not by an effect on white adipocyte cell number. Decreases in the serum lipid parameters, cholesterol and triglycerides are in line with decreases in body weight and fat pad mass. In females, a hierarchy in sensitivity to parameters could be deduced, where serum triglycerides are already affected at the lowest exposure levels (BMDL = 6.2 µg/kg bw/d), followed by a reduction in fat mass (BMDL = 65 µg/kg bw/d) and finally a lower body weight (BMDL = 849 µg/kg bw/d for week 27). These changes in lipid metabolism observed in our study could fit with PPARα activation (Martin et al. 2007; Rosen et al. 2007).

Other metabolic and energy balance-related parameters showed no effect and thus do not contribute to the change in body weight. In contrast, Hines et al. (2009) found an increase in insulin and leptin levels (at 21–33 weeks), coinciding with the increase in body weight, but similar to our study, no effects on glucose tolerance were observed. Differences between our and other developmental studies, especially with a similar dose range (Hines et al. 2009; Macon et al. 2011), can be explained by differences in experimental conditions. For example, we applied a three times longer exposure than Hines et al. (2009) and Macon et al. (2011), potentially also leading to a different total dose.

We observed an increase in relative liver weights in the adult F1 animals, whereas other developmental studies reported liver effects at an early age without a follow-up into adulthood (Wolf et al. 2007; Yang et al. 2009), or during adulthood, the effect did not exist anymore (Hines et al. 2009; Macon et al. 2011; White et al. 2009). The nearly significant histopathological changes in PFOA-exposed animals (increase in eosinophilic alteration, nuclear dysmorphology) are generally considered as age-related non-specific alterations in the liver (Thoolen et al. 2010), suggesting that PFOA affected the liver in a non-specific way.

The study design we applied mimics the continuous, dietary exposure of humans to PFOA (EFSA 2012). In addition, we used multiple low doses in a wide range to more accurately predict the shape of the dose–responses and calculate a BMD. We discontinued exposure after weaning to focus on the early life stage and explore our programming hypothesis. In contrast to the elimination half-life of 4 years in humans (Olsen et al. 2009), the elimination half-life in adult mice is 17 days, ensuring that our study focused primarily on exposure to PFOA during the early sensitive period of life (2 weeks pre-mating through to PND21) and the long-term consequences of this exposure. Moreover, due to the short half-life, a steady-state level of PFOA in serum of mice is already reached within 1 week (Lau et al. 2006). Accordingly, in a developmental study from Wolf et al. (2007), levels in offspring lowered 40 times within 6 weeks after maternal exposure was stopped. Therefore, it is unlikely that 23–25 weeks after termination of the actual exposure, retained PFOA is still active and the observed effects are a result from direct toxicity. However, bioaccumulation in and slow release from bones, similar to perfluorooctanesulfonic acid (Borg 2013), cannot be excluded. PFOA thus most likely exerted its effect during the exposure phase early in life, and the observed effects are a result of disrupted programming of metabolic homeostasis. Specifically, the latent effects on liver weight and fat pads and serum lipids in both sexes indicate a role for programming, since Xie et al. (2003) showed in adult male mice that decreases in body weight, white adipose mass and serum cholesterol return to normal levels after cessation of high PFOA exposure.

Programming could also explain differences in observed effects between male and female offspring. Programming probably comprises a complex of (epigenetic) mechanisms, including endocrine pathways, and sexes can respond differently or differ in sensitivity, resulting in diverse phenotypic outcomes. PFOA is a suspected endocrine disruptor (Du et al. 2013), supported by specific effects of developmental exposure to PFOA, which is known to affect female reproductive tissue and alter steroid hormone levels (Dixon et al. 2012; White et al. 2011a; Zhao et al. 2012). Such sexual dimorphic effects were not studied by others, since most developmental studies follow only female offspring into adulthood.

The current TDI for PFOA is established at 1.5 μg/kg bw/d based on a BMDL10 of 300 µg/kg bw/d for liver effects in rodents (EFSA 2008). Most of our effects showed BMDLs higher than 300 µg/kg bw/d, which is in line with the developmental study from Lau et al. (2006). The BMDL for the most sensitive effect in our study, i.e., decreased serum triglycerides, is 6.2 µg/kg bw/d. Because this parameter has borderline informative value (BMDU/BMDL = 100), the next lowest effect, the BMDL for perirenal fat pad weight in females at 65 µg/kg bw/d could be more relevant. Correction for the measured levels of PFOA in the feed, which were 15–30 % lower than nominal concentrations, leads to a BMDL of approximately 46–55 µg/kg bw/d. This developmental BMDL of 46 µg/kg bw/d is more than 10 times lower than the most sensitive developmental BMDL of 616 µg/kg bw/d established by Lau et al. (2006) and 6.5 times lower than the BMDL10 of 300 µg/kg bw/d used for the TDI. Applying our developmental BMDL of 46 µg/kg bw/d would lower the TDI to 230 ng/kg bw/d. However, this level is still a factor 15 higher than the human dietary exposure range of 0.16–15 ng/kg bw/d in infants (EFSA 2012).

In conclusion, our study with perinatal exposure to PFOA in mice produced metabolic effects in offspring, which were more pronounced in females. This is most likely due to disrupted programming of metabolic homeostasis, but the assayed endpoints did not provide a mechanistic explanation. The BMDL of effects in our study is below the known BMDL for developmental toxicity and also below the lowest BMDL used as the basis for the current TDI established by EFSA.

## Electronic supplementary material

Below is the link to the electronic supplementary material.

**Supplemental Fig.** **1 a** Perigonadal and **b** perirenal fat pad weights of females after perinatal PFOA exposure. C57BL/6JxFVB hybrid mice were perinatally exposed via maternal diet to 0–3000 µg/kg body weight/day (µg/kg bw/d) PFOA during gestation and lactation. For perirenal fat pad weight, the BMDL is 65 µg/kg bw/d. Explanation of the dose–response graph is in Fig. 2 legend. (EPS 1159 kb)

**Supplemental Fig.** **2** **a** Cholesterol and **b** triglyceride serum levels in females after perinatal PFOA exposure. C57BL/6JxFVB hybrid mice were perinatally exposed via maternal diet to 0–3000 µg/kg body weight/day (µg/kg bw/d) PFOA during gestation and lactation. BMDLs were 402 µg/kg bw/d for cholesterol and 6.2 µg/kg bw/d for triglyceride serum levels. Explanation of the dose–response graph is in Fig. 2 legend. (EPS 1127 kb)
